# Dr. Henry O. Walker

**Published:** 1912-05

**Authors:** 


Dr. Henry O. Walker of Detroit, died April 5, 1912 of pneumonia, aged 68. He was educated in his native city, Detroit, and at Albion College and the University of Michigan. He graduated in medicine at the Bellevue Hospital Medical College in 1867. In 1869, he edited the Detroit Review of Medicine, now defunct. He was twice Vice-President of the A. M. A., and was a founder and President of the Mississippi Valley Medical Association. He was one of the pioneer surgeons of the modern school in the middle west and was particularly well known as a writer on Genito-Urinary subjects.

